# Different associations of tumor *PIK3CA* mutations and clinical outcomes according to aspirin use among women with metastatic hormone receptor positive breast cancer

**DOI:** 10.1186/s12885-020-06810-8

**Published:** 2020-04-23

**Authors:** Anne Marie McCarthy, Nitya Pradeep Kumar, Wei He, Susan Regan, Michaela Welch, Beverly Moy, A. John Iafrate, Andrew T. Chan, Aditya Bardia, Katrina Armstrong

**Affiliations:** 1 Department of Medicine, Massachusetts General Hospital and Harvard Medical School, Boston, USA; 2 Department of Biostatistics, Epidemiology, and Informatics, University of Pennsylvania Perelman School of Medicine, 833 Blockley Hall, 423 Guardian Drive, Philadelphia, PA 19104 USA; 3 Harvard T H Chan School of Public Health, Boston, USA; 4 Massachusetts General Hospital Cancer Center, Harvard Medical School, Boston, USA; 5 Department of Pathology, Massachusetts General Hospital and Harvard Medical School, Boston, USA; 6 Clinical and Translational Epidemiology Unit, Massachusetts General Hospital and Harvard Medical School, Boston, USA

**Keywords:** Breast cancer, Aspirin, Metastasis, *PIK3CA*

## Abstract

**Introduction:**

The relationships among *PIK3CA* mutations, medication use and tumor progression remains poorly understood. Aspirin use post-diagnosis may modify components of the PI3K pathway, including AKT and mTOR, and has been associated with lower risk of breast cancer recurrence and mortality. We assessed time to metastasis (TTM) and survival with respect to aspirin use and tumor *PIK3CA* mutations among women with metastatic breast cancer.

**Methods:**

Patients with hormone receptor positive, HER2 negative (HR+/HER2-) metastatic breast cancer treated in 2009–2016 who received tumor genotyping were included. Aspirin use between primary and metastatic diagnosis was extracted from electronic medical records. TTM and survival were estimated using Cox proportional hazards regression.

**Results:**

Among 267 women with metastatic breast cancer, women with P*IK3CA* mutated tumors had longer TTM than women with *PIK3CA* wildtype tumors (7.1 vs. 4.7 years, *p* = 0.008). There was a significant interaction between *PIK3CA* mutations and aspirin use on TTM (*p* = 0.006) and survival (*p* = 0.026). *PIK3CA* mutations were associated with longer TTM among aspirin non-users (HR = 0.60 95% CI:0.44–0.82 *p* = 0.001) but not among aspirin users (HR = 1.57 0.86–2.84 *p* = 0.139). Similarly, *PIK3CA* mutations were associated with reduced mortality among aspirin non-users (HR = 0.70 95% CI:0.48–1.02 *p* = 0.066) but not among aspirin users (HR = 1.75 95% CI:0.88–3.49 *p* = 0.110).

**Conclusions:**

Among women who develop metastatic breast cancer, tumor *PIK3CA* mutations are associated with slower time to progression and mortality only among aspirin non-users. Larger studies are needed to confirm this finding and examine the relationship among aspirin use, tumor mutation profile, and the overall risk of breast cancer progression.

## Background

Breast cancer is at the forefront of precision medicine, a paradigm that uses individual variation in genes, environment, and lifestyle to improve disease prevention, diagnosis and treatment [1–3]. With advances in next generation sequencing, the application of this paradigm in breast cancer has progressed from identification of hormone receptor and HER-2 expression to genomic profiling of tumors to assess a range of gene expression levels and identify mutations in specific genes that may drive tumor progression and offer targets for therapeutic intervention [4–6].

*PIK3CA* is the most common somatic mutation identified in breast cancer to date, with prevalence of 25–40% in most studies [7]. Preclinical data have demonstrated that mutations in helical and kinase domains lead to increased PI3K activity. Activation of PI3K has downstream effects on the AKT and mTOR pathways that control key steps in cancer progression including cell cycle and metabolism [8]. Despite the pre-clinical evidence that *PIK3CA* is an oncogene, clinical studies of the outcomes of breast cancer patients with and without *PIK3CA* mutations have had conflicting results, demonstrating worse outcomes among patients with tumor *PIK3CA* mutations, no differences in outcomes by mutation status, and, most recently, better outcomes for women with hormone receptor positive (HR+) tumors with *PIK3CA* mutations [7, 9, 10].

Given the variation in these results, it has become increasingly important to understand how the effect of mutations in *PIK3CA* may be modified by other factors, including medication use. Although several studies have now demonstrated that the presence of *PIK3CA* mutations modifies the effect of cancer therapies such as trastuzamab and letrozole [10, 11], the effect of other medications, including aspirin and other non-steroidal anti-inflammatory drugs (NSAIDS), remains understudied. Aspirin/NSAIDs inhibit the activity of the enzymes cyclooxygenase (COX) 1 and COX-2, which results in a reduction in the synthesis of prostaglandins, which are involved in cell proliferation, apoptosis, migration, and invasion [12–14]. Aspirin and NSAID use have been shown in both observational studies and randomized trials to reduce cancer incidence [15–18] and metastasis [19–21] and increase survival [21, 22], including among breast cancer patients [13, 21, 23–25].

The specific mechanisms by which aspirin improves cancer outcomes, particularly breast cancer survival, are not known, but one hypothesized mechanism is through interaction with the PI3K pathway. Cox-2 expression has been shown to increase activation of AKT, a downstream component of the PI3K pathway [13]. Aspirin, which inhibits Cox-2, may in turn reduce *PIK3CA*-mediated cellular proliferation. Additionally, aspirin may inhibit mTOR, another downstream PI3K component, which would similarly reduce cell proliferation [13]. Aspirin use has been shown in pre-clinical studies to decrease growth and viability of *PIK3CA* breast cancer cells in vitro and in vivo [26, 27]. In colorectal cancer, aspirin use has been shown to improve survival among *PIK3CA* mutated cancers, but not *PIK3CA* wild type tumors [28]. No studies have evaluated whether the effect of *PIK3CA* mutation status on breast cancer outcomes differs by aspirin use.

In this study, we assessed time to metastasis and survival according to use of medications between time of diagnosis and metastasis and *PIK3CA* tumor mutation status among women with HR+/HER2- breast cancer that had metastasized.

## Methods

### Study design and population

We retrospectively identified 762 women with metastatic breast cancer treated at MGH from 2009 to 2016 who received tumor sequencing through a high-throughput tumor genotyping assay (detailed below). Since 2009, patients with metastatic breast cancer at Massachusetts General Hospital have routinely been offered tumor genotyping as part of clinical care. From this group, we excluded women with hormone receptor negative (ER- and PR-) and/or HER2 positive breast cancer (*N* = 319), women with no evidence of use of standard endocrine therapy for their primary breast cancer (*N* = 129), women whose initial diagnosis was stage IV breast cancer (*N* = 36), and women who developed metastatic disease within 6 months of primary diagnosis (*N* = 11).(Supplemental Figure 1). This resulted in a study of population of 267. The study was approved by the Partners Healthcare institutional review board and informed consent for use of clinically obtained data, including tumor genotyping data, for research purposes was obtained from all participants.

### Tumor genotyping

Tumor sequencing was performed on DNA from formalin-fixed, paraffin-embedded tumor tissue using a high-throughput tumor genotyping assay. From 2009 to 2014, a single-base extension multiplex sequencing assay known as “SNaPshot”, was used to detect the 8 most common *PIK3CA* mutations [29]. Since 2014, a Next Generation Sequencing panel assay “Snapshot-NGS” has been used, with complete sequencing coverage of exons 2,5,7-8,10,14,19, and 21 [30]. Both assays have a mutant allele frequency limit of detection of approximately 5%.

Most patients had genotyping of their metastatic tumor, while 24% (*N* = 63) of patients had their primary tumor genotyped. However, there is generally high genomic concordance of somatic *PIK3CA* mutations between primary and metastatic tumors for breast and other cancers, from 75% to over 90% concordance depending on the number of genes accessed [31–35].

### Clinical characteristics and medication use

Dates of primary and metastatic diagnosis and clinical characteristics such as age, stage and treatment at primary diagnosis were manually abstracted from electronic medical records. In our study population, 203 (76%) had at least one clinical encounter in the time period between primary and metastatic diagnosis of whom 172 had at least one oncology encounter, while 45 had only other types of visits, including primary care, OBGYN, or other specialist visits. Use of aspirin and NSAIDs were ascertained from electronic medical records (EMR) by adapting an algorithm previously developed and validated for use in our health system EMR [36]. Medication use was extracted from coded fields encompassing both inpatient and outpatient prescription records. In addition, all EMR free text clinical notes were loaded into an SQL-Server database and searched for evidence of aspirin or NSAID use using a strategy that located the terms listed in Online Resource 1, and eliminated cases in which the surrounding text indicated non-use (eg, instructions to avoid use, misspellings). Patients were classified as aspirin or NSAID (A/N) users if they had at least 2 instances of A/Ns noted - either 2 prescriptions, notes with text for A/Ns on 2 or more days, or a combination of a prescription and a note on different days. A/N use was ascertained beginning 6 months post primary diagnosis through the date of metastatic diagnosis. We excluded the 6-month period following primary diagnosis to avoid capturing short-term A/N use related to initial surgery. Where available, aspirin dose was also abstracted. We validated a random sample of 10 A/N users and 10 non-users based on the algorithm with manual chart review and found good agreement (kappa = 0.90). In addition to aspirin and NSAIDs use, we extracted use of statin drugs and beta blockers from medication lists and prescription records. Patients with any record of statin or beta blocker use in the period 6 months post primary diagnosis through date of metastatic diagnosis were categorized as users of statins or beta blockers. We included statins and beta blockers in the analysis, as controls for cardiac medication use, to assess whether any observed association of aspirin/NSAIDs with cancer outcome was specific to those drugs, or an association of cardiovascular treatment more generally.

### Statistical analysis

**Fig. 1:**
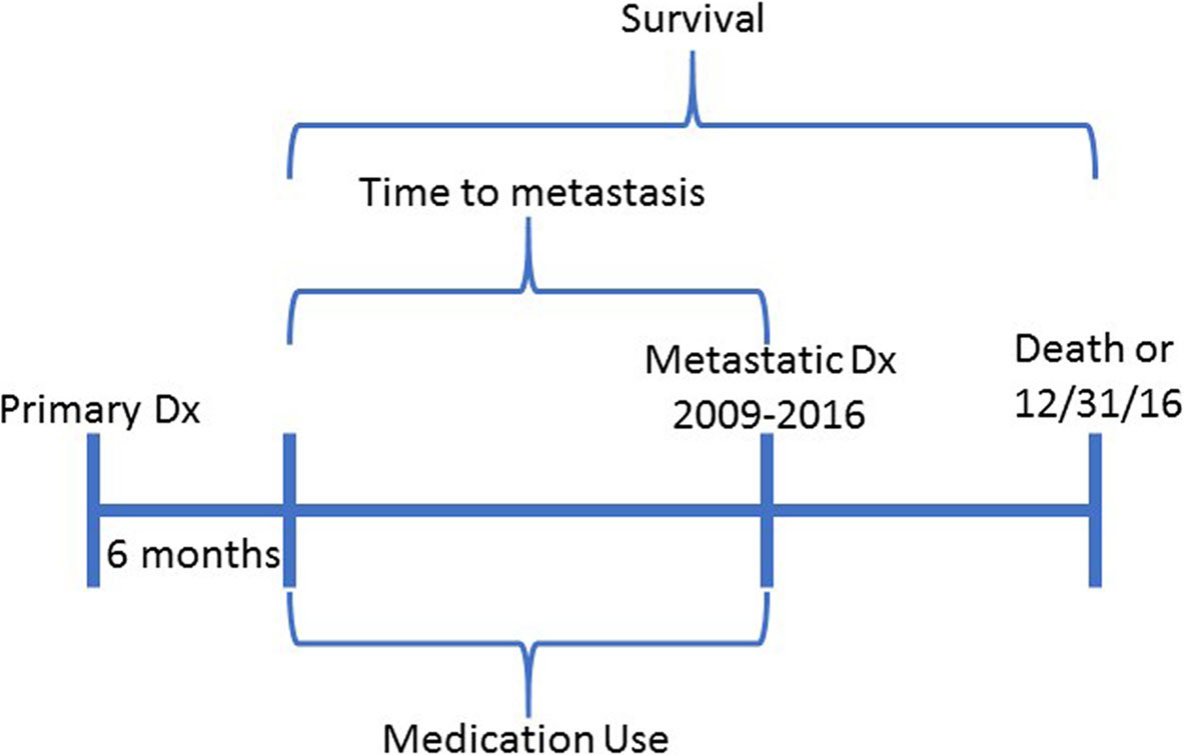
Study design and exposure assessment. Medication use was ascertained in the time period from 6 months after primary breast cancer diagnosis through metastatic diagnosis, which occurred from 2009 to 2016. Time to metastasis was calculated from 6 months following primary diagnosis through date of metastatic diagnosis. Survival time was calculated from 6 months following primary diagnosis through date of death or administrative censoring on December 31, 2016

Time to metastasis was calculated as time from 6 months post primary diagnosis through date of metastatic diagnosis, and survival time was calculated as time from 6 months post primary diagnosis through date of death or administrative censoring on December 31, 2016 (Fig. 1). Vital status and dates of death were ascertained from electronic medical records linked to the Social Security Death Master File. Differences in time to metastasis and survival time by medication use and *PIK3CA* status was assessed using Kaplan Meier survival analysis, log rank tests, life tables, and Cox proportional hazards modeling adjusted for age, stage, grade, ER and PR status, and use of chemotherapy at primary diagnosis. The interaction of *PIK3CA* mutation status and medication use was tested by including a cross product term in the models and statistical significance was evaluated using the Wald test. Linear combinations of HRs from the interaction models were calculated for the association of *PIK3CA* mutation status among medication users and non-users. In addition, we performed a sensitivity analysis excluding women with primary diagnosis before 2000 (Supplemental Table 2) and among women with at least one clinical encounter between primary and metastatic diagnosis (Supplemental Table 3). We assessed the proportional hazards assumption by examining Schoenfeld residuals. We assessed the associations of medications and *PIK3CA* status with early versus late metastasis defined as less than 5 years and greater than 5 years from primary diagnosis, adjusted for age, stage, grade, ER and PR status, and use of chemotherapy at primary diagnosis.

## Results

Descriptive characteristics of the study population are displayed in Table 1. Among the 267 women with metastatic disease, more than half received their primary diagnosis before age 50, with 20% diagnosed before age 40. Twenty-three percent were initially diagnosed with Stage I (*N* = 62) disease, 49% were diagnosed at Stage II, and 22% were diagnosed at Stage III. An additional 5% had an unknown stage at primary diagnosis. Most patients received chemotherapy for their primary tumor (80%). *PIK3CA* mutations were identified in 36% of patients with HR+/HER2- breast cancer. Twenty percent of patients were aspirin users, 32% used NSAIDs, and 40% used either/both types of drugs. Use of statins (13%) and betablockers (10%) was less common. Only 13 women had a record of aspirin use prior to their primary cancer diagnosis, and aspirin use did not differ by tumor genotype (data not shown).

**Table 1:** Characteristics of women with metastatic hormone receptor positive breast cancer and tumor genomic profile *N* = 267

	N	%
Age at Primary Dx		
< 40	54	20.2
40–49	101	37.8
50–59	69	25.8
≥ 60	43	16.1
Stage at Primary Dx		
1	62	23.2
2	131	49.1
3	59	22.1
Unknown	16	5.6
Grade at Primary Diagnosis		
1	26	9.7
2	135	50.6
3	81	30.3
9 (unknown)	25	9.4
ER +	262	98.1
PR+	218	81.7
Chemotherapy Use	214	80.1
Body Mass Index		
< 25	61	22.8
25–29.9	54	32.5
> =30	51	30.7
Missing	101	37.8
*PIK3CA* mutated	97	36.3
Medication use between primary and metastatic dx		
Aspirin	54	20.2
NSAIDs	85	31.8
Aspirin or NSAIDs	108	40.4
Statins	34	12.7
Betablockers	26	9.7
Typical Aspirin Dose among users		
325mg	15	27.8
81mg	28	51.8
Missing	11	20.4
Time to Metastasis		
< 5 years	129	48.3
5–10 years	66	24.7
≥ 10 years	72	27.0

**Fig. 2:**
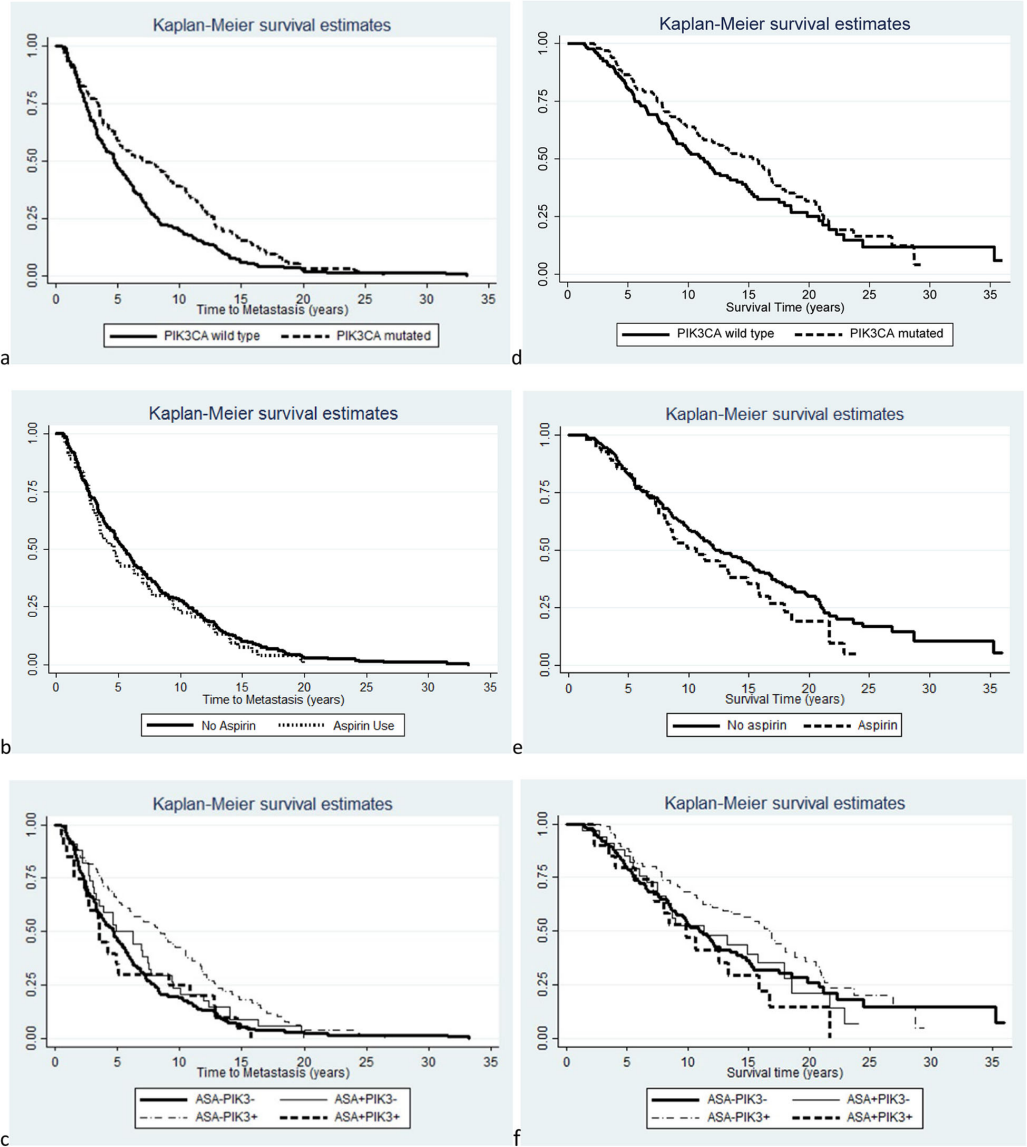
Kaplan-Meier analysis of time to metastasis (TTM) as displayed by *PIK3CA* mutation status (**a**); by aspirin use (**b**); and by combined aspirin use and *PIK3CA* mutation status (**c**). Kaplan-Meier analysis of time 6 months post primary diagnosis to death as displayed by *PIK3CA* mutation status (**d**); by aspirin use (**e**); and by combined aspirin use and *PIK3CA* mutation status (**f**)

The median TTM was 5.2 years (range 6 months-33 years). Time to metastasis is displayed by *PIK3CA* mutation status, by aspirin use, and by combined aspirin use and *PIK3CA* mutation status in Fig. 2. TTM was significantly longer for women with *PIK3CA* mutated tumors compared to women with *PIK3CA* wild type tumors (Median 7.1 vs. 4.7 yrs., *p* = 0.008). There was no difference in time to metastasis for aspirin users compared to non-users. Median TTM was longest for patients with *PIK3CA* mutated tumors who did not use aspirin (8.5 years), and women with *PIK3CA* mutated tumors who used aspirin had the shortest TTM (3.6 years, *p* = 0.008). The median time from 6 months post primary diagnosis to death was 9.6 years (range 1.4–36 years). There were no significant differences in survival time by *PIK3CA* status, aspirin use, or combined aspirin use and *PIK3CA* mutation status (Fig. 2). In addition, there were no statistically significant differences in TTM or survival for NSAIDS, beta blockers, or statins (data not shown). The distribution of time to metastasis and time to death by years from primary diagnosis are displayed using life table methods in Supplementary Tables 2 and 3.

Table 2 displays the results of Cox proportional hazards modeling of time to metastasis and survival time. *PIK3CA* mutated cancers had longer TTM compared to wild type tumors (HR = 0.73 95% CI 0.55–0.95 *p* = 0.022). Aspirin use was not associated with time to metastasis overall (HR = 0.91 95% CI 0.64–1.27 *p* = 0.592). Neither the frequency of documentation of aspirin use nor aspirin dose were significantly associated with time to metastasis (data not shown). However, we observed a significant interaction of *PIK3CA* mutation status and aspirin use on time to metastasis (p-interaction = 0.006). Among aspirin non-users, *PIK3CA* mutation was associated with longer TTM (HR = 0.60 95% CI 0.44–0.82 *p* = 0.001), but among aspirin users, *PIK3CA* mutation was not statistically significantly associated with TTM (OR = 1.57 95% CI 0.86–2.84 *p* = 0.139). Estimates of the associations of use of NSAIDs, combined aspirin and/or NSAIDs, statins and beta blockers with TTM are displayed in Table 3. There were no significant associations of use of NSAIDs alone, combined aspirin and/or NSAIDs, statins, or beta blockers with time to metastasis overall, and no significant interactions with *PIK3CA* tumor genotype (Table 3).

**Table 2:** Hazard ratios for metastasis and death by Aspirin use and tumor *PIK3CA* mutation among women with metastatic breast cancer (N = 267)

	N	%	Metastasis			Death		
			HR	95% CI	*p*-value	HR	95% CI	*p*-value
Age at Primary Dx								
< 40	54	20.2	1.00	Reference		1.00	Reference	
40–49	101	37.8	0.97	0.68–1.38	0.857	1.23	0.80–1.92	0.361
50–59	69	25.8	1.14	0.78–1.67	0.483	1.62	1.01–2.60	0.046
≥ 60	43	16.1	1.37	0.87–2.17	0.171	1.95	1.14–3.44	0.017
Stage at Primary Dx								
1	62	23.2	1.00	Reference		1.00	Reference	
2	131	49.1	1.41	1.01–1.94	0.040	1.34	0.89–1.95	0.149
3	59	22.1	1.53	1.04–2.20	0.026	1.59	1.01–2.45	0.041
Unknown	16	5.6	0.93	0.44–1.72	0.803	0.58	0.17–1.22	0.212
PR+	218	81.7	1.17	0.85–1.60	0.329	1.16	0.78–1.69	0.445
Grade								
1	26	9.7	1.00	Reference		1.00	Reference	
2	135	50.6	1.06	0.67–1.65	0.792	0.76	0.44–1.27	0.309
3	81	30.3	1.39	0.87–2.18	0.159	1.33	0.76–2.23	0.297
Unknown	25	9.4	0.51	0.28–0.93	0.025	0.44	0.21–0.93	0.027
Chemotherapy	214	80.1	0.98	0.70–1.36	0.886	1.15	0.77–1.72	0.497
*PIK3CA* mutated	97	36.3	0.73	0.55–0.95	0.022	0.86	0.61–1.18	0.376
Aspirin Use	54	20.2	0.91	0.64–1.27	0.592	1.04	0.68–1.54	0.855
			p-interaction		0.006			0.026
Aspirin non-user								
*PIK3CA* mutated	213	79.8	0.60	0.44–0.82	0.001	0.70	0.48–1.02	0.066
Aspirin user								
*PIK3CA* mutated	54	20.2	1.57	0.86–2.84	0.139	1.75	0.88–3.49	0.11

**Table 3:** Hazard ratios for metastasis and death by medication use and interaction with tumor *PIK3CA* mutation among women with metastatic breast cancer (*N* = 267)

	N	%	Multivariate ^a^			
			HR	95% CI	*p*-value	p-interaction^**b**^
**Metastasis**						
Aspirin	54	20.2	0.91	0.64–1.27	0.592	0.006
NSAIDs	85	31.8	0.86	0.65–1.14	0.293	0.516
Aspirin/NSAIDs	108	40.4	0.84	0.63–1.10	0.193	0.182
Statins	34	12.7	0.79	0.54–1.17	0.241	0.994
Beta Blockers	26	9.7	0.89	0.57–1.37	0.594	0.732
**Survival**						
Aspirin	54	20.2	1.04	0.68–1.54	0.855	0.026
NSAIDs	85	31.8	0.90	0.63–1.27	0.535	0.793
Aspirin/NSAIDs	108	40.4	0.90	0.64–1.25	0.525	0.131
Statins	34	12.7	0.99	0.63–1.57	0.980	0.570
Beta Blockers	26	9.7	1.25	0.76–2.04	0.385	0.544

^a^Models adjusted for age, stage, PR status, grade, chemotherapy use for primary diagnosis, *PIK3CA* mutation status

^b^p for interaction between medication use and *PIK3CA* mutation status

Neither *PIK3CA* mutation nor aspirin use were significantly associated with survival time (Table 2). However, similar to the TTM results, there was a significant interaction of *PIK3CA* mutation status and aspirin use with respect to survival time (Table 2, p-interaction = 0.026). The association of *PIK3CA* mutations with survival were not significant among aspirin users (HR = 0.70, 95% CI 0.48–1.02 *p* = 0.066) or non-users (HR = 1.75 95% CI 0.88–3.49 *p* = 0.110), however those associations were in opposite directions. There were no significant associations of use of NSAIDs alone, combined aspirin and/or NSAIDs, statins, or beta blockers with survival time overall and no significant interactions with *PIK3CA* tumor genotype (Table 3). We repeated the analyses of time to metastasis and survival excluding women with primary diagnosis prior to 2000, and among women with at least one clinical encounter between primary and metastatic diagnosis and the results were similar (Supplemental Tables 2 & 3). We did not observe any statistically significant deviations from the proportional hazards assumption based on Schoenfeld residuals in the Cox models.

Finally, we assessed the odds of metastasis within 5 years of primary diagnosis versus later metastasis (Table 4). Among this population of women with metastatic disease, there was no significant difference in early recurrence by *PIK3CA* mutation status or aspirin use, but the interaction of *PIK3CA* mutation status and aspirin use was significant (*p* = 0.026). Among aspirin non-users, *PIK3CA* mutation status was associated with lower risk of early recurrence (OR = 0.51 95% CI 0.27–0.94 *p* = 0.032) but among aspirin users, *PIK3CA* mutation status was associated with non-statistically significant increased risk of early recurrence (OR = 2.43 95% CI 0.72–8.21 *p* = 0.152).

**Table 4:** Odds of metastasis within 5 years of primary diagnosis

	N	%	Metastasis < 5 years		
			Multivariate		
			OR	95% CI	*p*-value
Age at Primary Dx					
< 40	54	20.2	1	Reference	
40–49	101	37.8	0.85	0.41–1.73	0.655
50–59	69	25.8	0.71	0.32–1.54	0.391
≥ 60	43	16.1	1.29	0.52–3.14	0.578
Stage at Primary Dx					
1	62	23.2	1.00	Reference	
2	131	49.1	1.38	0.72–2.64	0.321
3	59	22.1	2.93	1.34–6.37	0.007
Unknown	16	5.6	0.56	0.13–2.42	0.44
PR+	218	81.7	1.03	0.52–2.00	0.931
Grade					
1	26	9.7	1.00	Reference	
2	135	50.6	1.28	0.51–3.15	0.593
3	81	30.3	2.17	0.84–5.60	0.109
Unknown	25	9.4	0.44	0.11–1.65	0.222
Chemotherapy	214	80.1	1.08	0.55–2.11	0.826
*PIK3CA* mutated	97	36.3	0.71	0.41–1.22	0.213
Aspirin	54	20.2	1.31	0.67–2.55	0.421
p-interaction *PIK3CA* status & aspirin use					0.026
Aspirin non-user (*N* = 213)					
*PIK3CA* mutated^a^	213	79.8	0.51	0.27–0.94	0.032
Aspirin user (*N* = 54)					
*PIK3CA* mutated^a^	54	20.2	2.43	0.72–8.21	0.152

^a^Estimates obtained from logistic model including interaction term for medication use and *PIK3CA* mutation status

## Discussion

To our knowledge, this is the first study to assess the complex relationships among aspirin use, tumor *PIK3CA* mutation status and time to metastasis and survival among women who eventually developed metastatic disease. In this sample, we found that women with *PIK3CA* mutated cancers metastasized longer after initial presentation than women with *PIK3CA* wildtype tumors, but there was no significant difference in survival. Additionally, we identified a significant interaction of *PIK3CA* mutation status and aspirin use, whereby *PIK3CA* mutations were associated with longer time to metastasis and survival among aspirin non-users, but non-significant faster progression to metastatic disease and increased mortality risk among aspirin users. In contrast, there were no significant associations of NSAIDs, statins or beta blockers with time to metastasis or survival overall or significant interactions of these medications with *PIK3CA* mutation status. Given the difference between these results and the evidence from colorectal cancer, which suggests longer survival among patients with PIK3CA mutation who used aspirin, our study emphasizes the importance of further investigation of the complex effects of aspirin/NSAID use, tumor genomic subtypes and cancer progression, with both observational and randomized studies.

The current analysis focused on one part of the overall pathway by which aspirin/NSAIDs and PI3K may affect breast cancer outcomes - the time to metastasis and survival among women who eventually metastasize. It is important to emphasize that the study did not include women who did not metastasize, making it impossible to assess the impact of aspirin/NSAID use on risk of metastasis overall. However, these results add to the existing literature about outcomes among women with *PIK3CA* mutations at diagnosis by suggesting that, among women whose cancer metastasizes, the presence of a *PIK3CA* mutation may be associated with delayed time to metastasis. Our results are in line with those of a large pooled analysis that found *PIK3CA* mutations to be associated with longer invasive disease free survival [9]. Furthermore, the observed effect modification of *PIK3CA* mutation status by aspirin use highlights the important limitations to current knowledge about the tumor effects of aspirin. It is possible that aspirin use does suppress PI3K activity in breast cancer, but that this causes other, more aggressive pathways to predominate among women with *PIK3CA* mutated tumors who do metastasize. Our results are in contrast to evidence on colorectal cancer, which show benefits of aspirin for *PIK3CA* mutated tumors [28] and may suggest different biological pathways at work in breast cancer carcinogenesis. Alternatively, the benefits of aspirin use shown in previous studies may be due to effects on inflammation, platelets, or hormonal pathways in breast cancer [13], rather than *PIK3CA*. An analysis of the Nurses’ Health Study showed that aspirin reduced breast cancer mortality regardless of whether patients’ tumors expressed COX-2, suggesting that the mechanism of aspirin benefit was not via the COX-2 pathway [37]. These results highlight the complex nature of the interactions between medications and tumor genomics, which requires additional study to elucidate. Given the widespread use of aspirin, these findings need validation in additional datasets as it could have major public health implications.

The study has several strengths. The availability of tumor genetic profiles and detailed clinical data on medication use for a relatively large number of patients increased the power of the analysis. Natural language processing resulted in a high level of accuracy for the assessment of medication use compared to manual review of electronic medical records. In addition, the inclusion of statins and beta blockers increases the confidence that the observed association is not driven by an underlying risk of coronary heart disease (the primary indication for prescription of aspirin, statins and beta blockers) or level of adherence with preventive medications.

The results should be interpreted in light of several limitations. First, the study population was a sample of metastatic patients who had tumor genotyping during the course of clinical care, and we retrospectively assessed both time from primary diagnosis to metastasis as well as medication use. Therefore, our results may not be representative of all women with metastatic cancer, and importantly, does not include a key/valuable comparison group – women who did not develop metastatic cancer. Additionally, genotyping was primarily performed on metastatic rather than primary tumor samples due to sample availability. We expect based on prior studies [31–35] that *PIK3CA* mutation status would have been concordant between primary and metastatic tumors in the same patient, but more data is needed to confirm this. Additionally, we relied on retrospective assessment of medication use between primary cancer diagnosis and metastatic diagnosis from medical records. Since aspirin and NSAIDs are available over the counter, there is likely underreporting of medication used within this timeframe, leading to misclassification of exposure status, which may bias observed associations. Given these considerations, future prospective studies should ascertain over the counter medication use from multiple sources, including medical records and patient self-report. Additionally, though all patients received endocrine therapy, we did not have information on type of endocrine therapy, which may affect outcomes.

## Conclusions

Despite these limitations, our results are the first to assess the interplay of aspirin, *PIK3CA* tumor mutations, and outcomes, and can serve to inform further investigation of these associations. A large trial of aspirin use in the adjuvant setting is ongoing which randomizes aspirin use to women with HER2 negative, early stage breast cancers to assess recurrence risk by aspirin use (clinicaltrials.gov #NCT02927249). Our work suggests that looking at benefits by tumor genotype may be particularly valuable in weighing the risks and benefits of aspirin use among breast cancer patients, and highlights the role of precision medicine in the adjuvant setting. Better understanding of the complex interaction between epidemiological, clinical, and genomic factors in determining breast cancer outcomes could enable precision targeting of specific treatment strategies to reduce risks of recurrence and death.

## Supplementary information

**Additional file 1.**

## Data Availability

The data that support the findings of this study are available on request from the corresponding author [AMM]. The data are not publicly available due to fact that the data contains information that could compromise research participant privacy/consent.

## Abbreviations

A/N: Aspirin or NSAID
COX: Cyclooxygenase
ER: Estrogen receptor
HER2: Human epidermal growth factor 2
HR: Hazard ratio
HR +: hormone receptor positive
NSAIDS: Non-steroidal anti-inflammatory drugs
OR: Odds ratio
PR: Progesterone receptor
TTM: Time to metastasis
