# Supplementary material for: Different associations of tumor PIK3CA mutations and clinical outcomes according to aspirin use among women with metastatic hormone receptor positive breast cancer
Source: BMC Cancer. 2020 Apr 23;20:347. doi: 10.1186/s12885-020-06810-8 (PMC7181475; doi:10.1186/s12885-020-06810-8)
Supplement: Supplementary file 1 — Additional file 1. [file 12885_2020_6810_MOESM1_ESM.doc]

Supplementary Material for inclusion in manuscript submission to *BMC Cancer*

**Title:** **Association between tumor *PIK3CA* mutation, aspirin use and clinical outcomes in women with metastatic hormone receptor positive breast cancer**

Anne Marie McCarthy^1^, Nitya Pradeep Kumar^2^, Wei He^1^, Susan Regan^1^, Michaela Welch^1^, Beverly Moy^3^, A. John Iafrate^4^, Andrew T. Chan^5^, Aditya Bardia^3^, Katrina Armstrong^1^

1. Department of Medicine, Massachusetts General Hospital and Harvard Medical School, Boston, USA

2. Harvard T H Chan School of Public Health, Boston, USA

3. Massachusetts General Hospital Cancer Center, Harvard Medical School, Boston, USA

4. Department of Pathology, Massachusetts General Hospital and Harvard Medical School, Boston USA

5. Clinical and Translational Epidemiology Unit, Massachusetts General Hospital and Harvard Medical School, Boston, USA

**Corresponding author:**

Anne Marie McCarthy, ScM PhD

Division of General Internal Medicine

Massachusetts General Hospital

100 Cambridge Street, 16th Floor

Boston, MA 02114

amccarthy8@partners.org

tel 617-643-0679

Assessed for eligibility, metastatic breast cancer patients with tumor genotyping (n=762)

Excluded (n=495)

- Primary tumor ER and PR-, HER2+, or unknown (n=319)
- No evidence of endocrine therapy (n=129)
- Initially diagnosed with Stage IV breast cancer (n=36)
- Diagnosed with metastatic disease within 6 months of primary (n=11)

Analytic Sample (n=267)

**Supplemental Figure 1**

| **Supplemental Table 1: Lists of Natural Language Processing terms used to extract medication use from EMR** | | | | |
| --- | --- | --- | --- | --- |
| **Aspirin** | **NSAID** | | **Statins** | **Beta Blockers** |
| acetylsalicylic acid | Actron | Meclomen | Advicor | Acebutolol |
| Acuprin | Advil | Mefenamic acid | Altoprev | Atenolol |
| Aggrenox | Aleve | Meloxicam | Atorvastatin | Atenolol-Chlorthalidone |
| Anacin | Amigesic | Mobic | Atrovastatin-Amlodipine | Betapace |
| asa | Anaflex 750 | Mobidin | Caduet | Betapace AF |
| Ascriptin | Anaprox | Mobogesic | Crestor | Betaxolol |
| Aspercin | Ansaid | Mono-Gesic | Fluvastatin | Bisoprolol |
| Aspergum | Arthritab | Motrin | Lescol | Bisoprolol-HTC |
| Aspirin (Acetylsalicylic Acid) | Arthropan | Motrin IB | Lescol XL | Blocadren |
| Aspirin Buffered | Arthrotec | Nabumetone | Lipitor | Brevibloc |
| Aspirin Controlled Release | Bayer Select | Nalfon | Lovastatin | Carteolol |
| Aspirin Enteric Coated | Bextra | Naprelan | Lovastatin-Niacin | Cartrol |
| Aspirin+Butalbital | Cataflam | Naprosyn | Mevacor | Carvedilol |
| Aspirin+Caffeine | Celebrex | Naproxen | Pravachol | Coreg |
| Aspirin+Dipyridamole | Celecoxib | Naproxen sodium | Pravastatin | Coreg CR |
| Aspirin+Oxycodone | Choline salicylate | Nuprin | Rosuvastatin | Corgard |
| Aspirtab | Clinoril | Orudis | Simvastatin | Corzide |
| Bayer | CMT | Orudis KT | Simvastatin and Ezetimibe | Esmolol |
| Bufferin | Daypro | Oruvail | Vytorin | Inderal |
| Ecotrin | Diclofenac potassium | Oxaprozin | Zocor | Inderal LA |
| Genacote | Diclofenac sodium | Piroxicam |  | Inderide |
| St Joseph | Diflunisal | Ponstel |  | Innopran XL |
| aspirin (including misspellings such as 'asperin' and 'aspirn') | Disalcid | Relafen |  | Kerlone |
| Aspirin baby (Acetylsalicylic Acid (Children’s)) | Doan's Pills | Rofecoxib |  | Labetalol |
| Aspirin Enteric Coated (Children’s) | Dolobid | Salflex |  | Levatol |
| Enteric Coated Aspirin (Aspirin Enteric Coated) | Etodolac | Salsalate |  | Lopressor |
| Aspirin/Caffeine/Dihydrocodeine (Synalgos-DC) | Feldene | Salsitab |  | Metoprolol |
| Aspirin+Caffeine+Dihydrocodeine | Fenoprofen calcium | Sodium salicylate |  | Nadolol |
|  | Flurbiprofen | Sulindac |  | Penbutolol |
|  | Ibuprofen | Tolectin |  | Pindolol |
|  | Indocin | Tolmetin sodium |  | Propanolol |
|  | Indocin SR | Tricosal |  | Propanolol-HCT |
|  | Indomethacin | Trilisate |  | Sectral |
|  | Ketoprofen | Valdecoxib |  | Sotalol |
|  | Lodine | Vioxx |  | Tenoretic |
|  | Lodine XL | Voltaren |  | Tenormin |
|  | Magan | Voltaren XR |  | Timolol |
|  | Meclofenamate sodium Marthritic | Magnesium salicylate  Diclofenac sodium with misoprostol |  | Toprol XL |
|  | Choline and magnesium salicylates |  |  | Trandate Normodyne |
|  |  |  |  | Visken |
|  |  |  |  | Zebeta |
|  |  |  |  | Ziac |
|  |  |  |  | Nadolol-Bendroflumethiazide |

| Supplemental Table 2: Time to Metastasis by *PIK3CA* genotype and Aspirin use | | | | | | | | |  |  |
| --- | --- | --- | --- | --- | --- | --- | --- | --- | --- | --- |
|  | *PIK3CA* wildtype | | | | | *PIK3CA* mutant | | | | |
| Time to Metastasis | Deaths | Lost | Cumulative Rate | 95% CI | | Deaths | Lost | Cumulative Rate | 95% CI | |
| 0-2 years | 31 | 0 | 0.18 | 0.13 | 0.25 | 16 | 0 | 0.16 | 0.10 | 0.26 |
| 2-5 years | 58 | 0 | 0.52 | 0.45 | 0.60 | 24 | 0 | 0.41 | 0.32 | 0.52 |
| 5-10 years | 47 | 0 | 0.80 | 0.74 | 0.86 | 19 | 0 | 0.61 | 0.51 | 0.71 |
| 10-15 years | 24 | 0 | 0.94 | 0.90 | 0.97 | 23 | 0 | 0.85 | 0.77 | 0.91 |
| ≥15 years | 10 | 0 | 1.00 |  |  | 15 | 0 | 1.00 |  |  |
|  |  |  |  |  |  |  |  |  |  |  |
|  | Asprin non-user | | | | | Aspirin user | | | | |
| Time to Metastasis | Deaths | Lost | Cumulative Rate | 95% CI | | Deaths | Lost | Cumulative Rate | 95% CI | |
| 0-2 years | 38 | 0 | 0.18 | 0.13 | 0.24 | 9 | 0 | 0.17 | 0.09 | 0.30 |
| 2-5 years | 61 | 0 | 0.46 | 0.40 | 0.53 | 21 | 0 | 0.56 | 0.43 | 0.69 |
| 5-10 years | 55 | 0 | 0.72 | 0.66 | 0.78 | 11 | 0 | 0.76 | 0.64 | 0.86 |
| 10-15 years | 38 | 0 | 0.90 | 0.86 | 0.94 | 9 | 0 | 0.93 | 0.84 | 0.98 |
| ≥15 years | 21 | 0 | 1.00 |  |  | 4 | 0 | 1.00 |  |  |
|  |  |  |  |  |  |  |  |  |  |  |
|  | Aspirin Non-user/*PIK3CA* Wild type | | | | | Aspirin user/*PIK3CA* Wild type | | | | |
| Time to Metastasis | Deaths | Lost | Cumulative Rate | 95% CI | | Deaths | Lost | Cumulative Rate | 95% CI | |
| 0-2 years | 27 | 0 | 0.20 | 0.14 | 0.28 | 4 | 0 | 0.12 | 0.05 | 0.28 |
| 2-5 years | 45 | 0 | 0.53 | 0.45 | 0.62 | 13 | 0 | 0.50 | 0.35 | 0.68 |
| 5-10 years | 38 | 0 | 0.81 | 0.74 | 0.87 | 9 | 0 | 0.76 | 0.61 | 0.89 |
| 10-15 years | 19 | 0 | 0.95 | 0.90 | 0.98 | 5 | 0 | 0.91 | 0.79 | 0.98 |
| ≥15 years | 7 | 0 | 1.00 |  |  | 3 | 0 | 1.00 |  |  |
|  |  |  |  |  |  |  |  |  |  |  |
|  | Aspirin Non-user/*PIK3CA mutant* | | | |  | Aspirin Non-user/*PIK3CA mutant* | | | |  |
| Time to Metastasis | Deaths | Lost | Cumulative Rate | 95% CI |  | Deaths | Lost | Cumulative Rate | 95% CI |  |
| 0-2 years | 11 | 0 | 0.14 | 0.08 | 0.24 | 5 | 0 | 0.25 | 0.11 | 0.50 |
| 2-5 years | 16 | 0 | 0.35 | 0.26 | 0.47 | 8 | 0 | 0.65 | 0.45 | 0.84 |
| 5-10 years | 17 | 0 | 0.57 | 0.46 | 0.68 | 2 | 0 | 0.75 | 0.55 | 0.91 |
| 10-15 years | 19 | 0 | 0.82 | 0.73 | 0.89 | 4 | 0 | 0.95 | 0.79 | 1.00 |
| ≥15 years | 14 | 0 | 1.00 |  |  | 1 | 0 | 1.00 |  |  |

| Supplemental Table 3: Time to Death by *PIK3CA* genotype and Aspirin use | | | | | | |  |  |  |  |
| --- | --- | --- | --- | --- | --- | --- | --- | --- | --- | --- |
|  | *PIK3CA* wildtype | | | | | *PIK3CA* mutant | | | | |
| Time to Death | Deaths | Lost | Rate | 95% CI | | Deaths | Lost | Rate | 95% CI | |
| 0-2 years | 4 | 0 | 0.02 | 0.01 | 0.06 | 0 | 0 | 0.00 | 0.00 | 0.00 |
| 2-5 years | 29 | 4 | 0.20 | 0.14 | 0.26 | 13 | 2 | 0.14 | 0.08 | 0.22 |
| 5-10 years | 41 | 23 | 0.47 | 0.39 | 0.55 | 21 | 3 | 0.36 | 0.27 | 0.47 |
| 10-15 years | 19 | 16 | 0.63 | 0.55 | 0.71 | 11 | 9 | 0.49 | 0.39 | 0.60 |
| ≥15 years | 16 | 18 | 0.87 | 0.78 | 0.93 | 23 | 15 | 0.88 | 0.78 | 0.94 |
|  |  |  |  |  |  | Aspirin user | | | | |
|  | Asprin non-user | | | | |
| Time to Death | Deaths | Lost | Rate | 95% CI |  | Deaths | Lost | Rate | 95% CI |  |
| 0-2 years | 3 | 0 | 0.01 | 0.00 | 0.04 | 1 | 0 | 0.02 | 0.00 | 0.12 |
| 2-5 years | 34 | 4 | 0.18 | 0.13 | 0.23 | 8 | 2 | 0.17 | 0.09 | 0.30 |
| 5-10 years | 46 | 20 | 0.41 | 0.35 | 0.48 | 16 | 6 | 0.50 | 0.37 | 0.65 |
| 10-15 years | 24 | 23 | 0.56 | 0.49 | 0.63 | 6 | 2 | 0.65 | 0.51 | 0.79 |
| ≥15 years | 31 | 28 | 0.86 | 0.79 | 0.92 | 8 | 5 | 0.92 | 0.79 | 0.98 |
|  |  |  |  |  |  |  |  |  |  |  |
|  | Aspirin Non-user/*PIK3CA* Wild type | | | | | Aspirin user/*PIK3CA* Wild type | | | | |
| Time to Death | Deaths | Lost | Rate | 95% CI |  | Deaths | Lost | Rate | 95% CI |  |
| 0-2 years | 3 | 0 | 0.02 | 0.01 | 0.07 | 1 | 0 | 0.03 | 0.00 | 0.19 |
| 2-5 years | 25 | 3 | 0.21 | 0.15 | 0.29 | 4 | 1 | 0.15 | 0.06 | 0.32 |
| 5-10 years | 31 | 18 | 0.46 | 0.38 | 0.55 | 10 | 5 | 0.48 | 0.32 | 0.67 |
| 10-15 years | 16 | 15 | 0.64 | 0.55 | 0.73 | 3 | 1 | 0.61 | 0.43 | 0.78 |
| ≥15 years | 11 | 14 | 0.86 | 0.76 | 0.93 | 5 | 4 | 0.89 | 0.71 | 0.98 |
|  |  |  |  |  |  |  |  |  |  |  |
|  | Aspirin Non-user/*PIK3CA mutant* | | | | | Aspirin Non-user/*PIK3CA mutant* | | | | |
| Time to Death | Deaths | Lost | Rate | 95% CI |  | Deaths | Lost | Rate | 95% CI |  |
| 0-2 years | 0 | 0 | 0 | 0 | 0 | 0 | 0 | 0 | 0 | 0 |
| 2-5 years | 9 | 1 | 0.12 | 0.06 | 0.21 | 4 | 1 | 0.21 | 0.08 | 0.46 |
| 5-10 years | 15 | 2 | 0.32 | 0.23 | 0.44 | 6 | 1 | 0.53 | 0.33 | 0.76 |
| 10-15 years | 8 | 8 | 0.44 | 0.33 | 0.56 | 3 | 1 | 0.72 | 0.50 | 0.90 |
| ≥15 years | 20 | 14 | 0.85 | 0.74 | 0.93 | 3 | 1 | 0.96 | 0.75 | 1.00 |

| **Table 2: Metastasis and survival by Aspirin use and tumor *PIK3CA* mutation among ER/PR+HER2- metastatic breast cancer patients with primary cancer was diagnosed after 2000 (N=203)** | | | | | | |
| --- | --- | --- | --- | --- | --- | --- |
|  | Metastasis | | | Survival | | |
|  | HR | 95% CI | p-value | HR | 95% CI | p-value |
| Age at Primary Dx | | | | | | |
| <40 | 1.00 | Reference |  | 1.00 | Reference |  |
| 40-49 | 0.70 | 0.45-1.08 | 0.111 | 1.14 | 0.66-1.95 | 0.639 |
| 50-59 | 0.93 | 0.58-1.45 | 0.736 | 1.54 | 0.88-2.70 | 0.130 |
| ≥60 | 0.83 | 0.49-1.38 | 0.466 | 1.58 | 0.85-2.89 | 0.143 |
| Stage at Primary Dx | | | | | | |
| 1 | 1.00 | Reference |  | 1.00 | Reference |  |
| 2 | 1.19 | 0.81-1.75 | 0.368 | 1.09 | 0.68-1.72 | 0.719 |
| 3 | 1.88 | 1.21-2.92 | 0.005 | 2.06 | 1.21-3.49 | 0.007 |
| Unknown | 0.61 | 0.25-1.49 | 0.281 | 0.41 | 0.13-1.28 | 0.125 |
| PR+ | 0.97 | 0.67-1.40 | 0.874 | 0.90 | 0.57-1.42 | 0.660 |
| Grade | | | | | | |
| 1 | 1.00 | Reference |  | 1.00 | Reference |  |
| 2 | 1.56 | 0.93-2.63 | 0.088 | 1.00 | 0.56-1.80 | 0.997 |
| 3 | 2.75 | 1.57-4.80 | <0.001 | 2.24 | 1.19-4.21 | 0.012 |
| Unknown | 2.38 | 1.00-5.67 | 0.050 | 1.55 | 0.59-4.05 | 0.373 |
| Chemotherapy | 0.98 | 0.66-1.45 | 0.919 | 1.18 | 0.72-1.92 | 0.518 |
| *PIK3CA* mutated | 0.80 | 0.57-1.10 | 0.172 | 1.06 | 0.72-1.55 | 0.750 |
| Aspirin Use | 1.05 | 0.73-1.50 | 0.795 | 1.09 | 0.71-1.68 | 0.680 |
|  | p-interaction |  | 0.031 |  |  | 0.158 |
| Aspirin non-user (N=159) | | | | | | |
| *PIK3CA* mutated | 0.65 | 0.44-0.96 | 0.028 | 0.90 | 0.58-1.41 | 0.659 |
| Aspirin user (N=44) | | | | | | |
| *PIK3CA* mutated | 1.49 | 0.78-2.84 | 0.220 | 1.70 | 0.81-3.57 | 0.162 |

| **Table 3: Metastasis and survival by Aspirin use and tumor *PIK3CA* mutation among ER/PR+HER2- metastatic breast cancer patients with primary cancer was diagnosed in 2000 or after (N=203)** | | | | | | |
| --- | --- | --- | --- | --- | --- | --- |
|  | Metastasis | | | Survival | | |
|  | HR | 95% CI | p-value | HR | 95% CI | p-value |
| Age at Primary Dx | | | | | | |
| <40 | 1.00 | Reference |  | 1.00 | Reference |  |
| 40-49 | 1.02 | 0.67-1.58 | 0.898 | 1.31 | 0.77-2.23 | 0.323 |
| 50-59 | 1.36 | 0.84-2.19 | 0.207 | 2.25 | 1.25-4.06 | 0.007 |
| ≥60 | 1.50 | 0.86-2.59 | 0.150 | 2.74 | 1.40-5.40 | 0.003 |
| Stage at Primary Dx | | | | | | |
| 1 | 1.00 | Reference |  | 1.00 | Reference |  |
| 2 | 1.49 | 1.03-2.17 | 0.036 | 1.30 | 0.82-2.05 | 0.266 |
| 3 | 1.88 | 1.23-2.88 | 0.004 | 1.63 | 0.98-2.70 | 0.062 |
| Unknown | 1.65 | 0.66-4.12 | 0.279 | 1.12 | 0.36-3.44 | 0.843 |
| PR+ | 1.09 | 0.75-1.60 | 0.649 | 1.13 | 0.72-1.76 | 0.599 |
| Grade | | | | | | |
| 1 | 1.00 | Reference |  | 1.00 | Reference |  |
| 2 | 1.01 | 0.62-1.66 | 0.960 | 0.70 | 0.38-1.27 | 0.236 |
| 3 | 1.52 | 0.90-2.55 | 0.117 | 1.69 | 0.91-3.12 | 0.097 |
| Unknown | 0.38 | 0.17-0.84 | 0.017 | 0.35 | 0.13-0.93 | 0.036 |
| Chemotherapy | 0.97 | 0.67-1.41 | 0.884 | 1.33 | 0.83-2.13 | 0.232 |
| *PIK3CA* mutated | 0.74 | 0.54-1.00 | 0.053 | 0.86 | 0.59-1.24 | 0.414 |
| Aspirin Use | 0.92 | 0.63-1.32 | 0.637 | 0.96 | 0.62-1.49 | 0.846 |
|  | p-interaction |  | 0.002 |  |  | 0.004 |
| Aspirin non-user (N=149) | | | | | | |
| *PIK3CA* mutated | 0.53 | 0.36-0.77 | 0.001 | 0.59 | 0.37-0.94 | 0.026 |
| Aspirin user (N=54) | | | | | | |
| *PIK3CA* mutated | 1.77 | 0.97-3.23 | 0.065 | 2.11 | 1.04-4.26 | 0.037 |
